# Polygenic overlap of substance use behaviors and disorders with externalizing and internalizing problems independent of genetic correlations

**DOI:** 10.1017/S0033291725000108

**Published:** 2025-03-31

**Authors:** Laila Al-Soufi, Guy Hindley, Linn Rødevand, Alexey A. Shadrin, Piotr Jaholkowski, Vera Fominykh, Romain Icick, Markos Tesfaye, Javier Costas, Ole A. Andreassen

**Affiliations:** 1 Psychiatric Genetics group, Instituto de Investigación Sanitaria de Santiago de Compostela (IDIS), Santiago de Compostela, Galicia, Spain. Red de Investigación en Atención Primaria de Adicciones (RIAPAd); 2 Department of Zoology, Genetics and Physical Anthropology, Universidade de Santiago de Compostela (USC), Santiago de Compostela, Galicia, Spain; 3 Centre for Precision Psychiatry, Division of Mental Health and Addiction, Institute of Clinical Medicine, University of Oslo, Oslo, Norway; 4 Université Paris-Cité, INSERM, Optimisation thérapeutique en neuropsychopharmacologie OPTEN U1144, 75006, Paris, France; 5 Department of Clinical Science, University of Bergen, Bergen, Norway; 6 Complexo Hospitalario Universitario de Santiago de Compostela (CHUS), Servizo Galego de Saúde (SERGAS), Santiago de Compostela, Galicia, Spain

**Keywords:** shared genetic susceptibility, alcoholism, nicotine dependence, alcohol use disorder, alcohol consumption, tobacco use disorder, polygenicity, genetic correlation, psychiatric genetics, externalizing problems, internalizing problems

## Abstract

**Background:**

Externalizing and internalizing pathways may lead to the development of substance use behaviors (SUBs) and substance use disorders (SUDs), which are all heritable phenotypes. Genetic correlation studies have indicated differences in the genetic susceptibility between SUBs and SUDs. We investigated whether these substance use phenotypes are differently related to externalizing and internalizing problems at a genetic level.

**Methods:**

We analyzed data from genome-wide association studies (GWAS) of four SUBs and SUDs, five externalizing traits, and five internalizing traits using the bivariate causal mixture model (MiXeR) to estimate genetic overlap beyond genetic correlation.

**Results:**

Two distinct patterns were found. SUBs demonstrated high genetic overlap but low genetic correlation of shared variants with internalizing traits, suggesting a pattern of mixed effect directions of shared genetic variants. Conversely, SUDs and externalizing traits exhibited considerable genetic overlap with moderate to high positive genetic correlation of shared variants, suggesting concordant effect direction of shared risk variants.

**Conclusions:**

These results highlight the importance of the externalizing pathway in SUDs as well as the limited role of the internalizing pathway in SUBs. As MiXeR is not intended for the identification of specific genes, further studies are needed to reveal the underlying shared mechanisms of these traits.

## Introduction

Substance use stands as a major risk factor contributing significantly to the global burden of disease (Degenhardt et al., 2018). To improve prevention and treatment, it is important to increase our understanding of the pathogenesis of substance use behaviors (SUBs) (e.g. initiation, lifetime use, and severity of consumption) and substance use disorders (SUDs). This includes identifying factors influencing different aspects of substance use, from severity of substance consumption to pathological substance use.

Evidence from developmental psychology suggests two main pathways for substance use initiation and escalation towards SUDs: an *externalizing* pathway characterized by high levels of impulsivity, aggression, and sensation-seeking, and an *internalizing* pathway characterized by using substances to cope with negative affect, depression, and anxiety (Hussong, Jones, Stein, Baucom, & Boeding, 2011; Krueger et al., 2002). This is supported by prospective evidence of distinct externalizing and internalizing pathways leading to future SUBs and SUDs (Meque, Dachew, Maravilla, Salom, & Alati, 2019; Ning, Gondek, Patalay, & Ploubidis, 2020). However, evidence is weaker for the internalizing pathway. This may be due to comorbidity between internalizing and externalizing problems and differences regarding gender, developmental timing, or type of substance use (Heitzeg, Hardee, & Beltz, 2018; Ning et al., 2020). For instance, internalizing traits seem to be related to reduced alcohol consumption in middle adulthood subjects, but to increased consumption in more severe outcomes (i.e. heavy drinking and alcohol use disorder [AUD]) during transition to adulthood (Ning et al., 2020).

Additional support for a causal effect of both externalizing and internalizing problems on SUBs and SUDs comes from the Mendelian randomization studies. Mendelian randomization leverages information from molecular genetic studies to infer causal relationships between phenotypes (Chen, Tubbs, Liu, Thach, & Sham, 2024). This approach found evidence for a causal effect of attention-deficit/hyperactivity disorder (ADHD) on tobacco and cannabis use behaviors, but also for reverse causation (Treur et al., 2019; Vilar-Ribó et al., 2020). It also indicated that depression and neuroticism are risk factors for tobacco use and alcohol dependence and discarded reverse causation (Polimanti et al., 2019; Sallis, Davey Smith, & Munafò, 2019; Yao et al., 2021). However, there is also some evidence for a bidirectional causal relationship between depression and smoking behavior (Wootton et al., 2020).

Twin and family studies have estimated heritability of around 40–60% for different SUDs, partly specific and partly shared between substances (Deak & Johnson, 2021). Genome-wide association studies (GWAS) revealed that a considerable portion of the heritability is due to the additive effect of many common variants of low individual effect, that is SUDs are highly polygenic traits. Many of these genetic variants are shared between SUDs and among SUDs and related phenotypes (Deak & Johnson, 2021; Gelernter & Polimanti, 2021). Polygenic scores (PGS) may be used to estimate the risk of SUD as the sum of susceptibility alleles carried by an individual weighted by their effect. Cross-trait PGS confirmed the existence of shared genetic susceptibility between related phenotypes. For instance, PGS of the average number of alcoholic drinks consumed per week (DrnkWk), major depressive disorder (MDD), and ADHD are associated with AUD (Facal et al., 2021), PGS of ADHD is associated with tobacco use (Leppert et al., 2020), and PGS of smoking initiation with DrnKWk, nicotine dependence and conduct disorder (Chang et al., 2019).

Among the different methods to measure shared genetic susceptibility, cross-trait Linkage Disequilibrium (LD) score regression has reached enormous popularity in recent years due to the main advantage that it relies only on GWAS summary statistics. LD score regression calculates the correlation coefficient of additive genetic effects for two traits across the genome (genetic correlation, r_g_) assuming a simple infinitesimal model, that is all SNPs are causal with normally distributed additive genetic effects (Bulik-Sullivan et al., 2015). Recent studies have examined the genetic correlation between different SUBs and SUDs using LD score regression. They have shown that smoking severity, defined as the number of cigarettes smoked per day (Cigsperday), and nicotine dependence are almost genetically identical (r_g_ = 0.95) (Quach et al., 2020). In fact, Cigsperday was proposed as a ‘genetic proxy’ for nicotine dependence, considering their strong genetic correlation and their similar pattern of associations with multiple diseases (Sanchez-Roige, Cox, Johnson, Hancock, & Davis, 2021). By contrast, the genetic correlation between alcohol consumption, measured as the number of drinks per week (DrnkWk), and alcohol dependence was weaker (r_g_ = 0.37) (Walters et al., 2018). Similarly, there is a moderate genetic correlation between smoking initiation and nicotine dependence (r_g_ = 0.40) (Quach et al., 2020). Low to moderate genetic correlation also exists between SUBs involving different substances, such as smoking initiation and DrnkWk (r_g_ = 0.27) (Saunders et al., 2022).

Significant genetic correlations have also been identified between both externalizing and internalizing traits and both SUBs and SUDs across different substances. For example, ADHD was genetically correlated with SUDs such as nicotine dependence, alcohol dependence and cannabis use disorder (CUD) (Abdellaoui, Smit, van den Brink, Denys, & Verweij, 2021; Vink, Treur, Pasman, & Schellekens, 2020; Walters et al., 2018); as well as with SUBs such as tobacco and cannabis use (Liu et al., 2019; Pasman et al., 2018; Soler Artigas et al., 2020). Other risky-behavior traits considered as externalizing were associated with SUBs. For example, age at first sexual intercourse (AFS) and age at first birth (AFB) showed a significant genetic correlation with age of smoking initiation and cannabis use (Mills et al., 2021). Further, other studies reported a significant genetic correlation of depression with both SUBs and SUDs, including smoking initiation, smoking cessation, nicotine dependence, and alcohol dependence (Abdellaoui et al., 2021; Liu et al., 2019; Vink et al., 2020; Walters et al., 2018).

Despite the insight provided by genome-wide genetic correlation to understand shared genetic susceptibility among related phenotypes, it has a clear limitation. Genome-wide genetic correlation, as estimated by LD score regression, can capture genetic overlap between two phenotypes only when the majority of shared variants exhibit a strong net correlation in the direction of effects relative to each other (i.e. effects are correlated in the same or opposite directions in the two phenotypes). However, it does not allow for the assessment of genetic overlap if the shared variants reveal a balanced mixture of concordant and discordant directions of effects. The bivariate causal mixture model (MiXeR) overcomes this limitation, characterizing the pattern of genetic overlap between two traits beyond genome-wide genetic correlation (Frei et al., 2019). First, MiXeR estimates the number of causal variants involved in each trait, instead of assuming an infinitesimal model. Next, MiXeR estimates the number of shared variants between the two traits and the genetic correlation of shared variants. Therefore, in contrast to genome-wide genetic correlation studies, MiXeR enables the discovery of genetic overlap even if the shared variants possess a mixture of concordant and discordant effect directions.

In the current study, we aimed to characterize the genetic relationship of SUBs and SUDs with externalizing and internalizing traits using MiXeR. Specifically, we investigated if: (i) SUBs and SUDs differ in their pattern of polygenic overlap with externalizing and internalizing traits, including the proportion of genetic overlap and genetic correlation of shared variants, and (ii) these differences are present regardless of the particular substance involved. This approach can improve our understanding of the contribution of the externalizing and internalizing pathways to SUBs and SUDs.

## Methods

### Genome-wide association studies (GWAS)

We included summary statistics for SUBs and SUDs and externalizing and internalizing traits from large GWAS (Table 1 and Supplementary Table 1). These GWAS were selected according to their large sample size and public availability. All GWAS summary statistics were based on samples of European ancestry to share similar linkage disequilibrium patterns, a requirement for MiXeR analysis.

**Table 1.** GWAS summary statistics used in MiXeR analysis

Trait	Description	Source	Sample size (N cases)
SUBs and SUDs or SUD-proxies			
EverSmk	Ever been a regular smoker, i.e. ever vs. never regular smokers	(Saunders et al., 2022)	805,431 (393,707)
Cigsperday	Average number of cigarettes smoked per day, either as a current or as a former smoker	(Saunders et al., 2022)	326,497
DrnkWk	Average number of drinks drunk each week	(Saunders et al., 2022)	666,978
AUD	Alcohol use disorder	(Icick et al., 2023)	981,384 (75,635)
Externalizing traits			
ADHD	Attention deficit hyperactivity disorder	(Demontis et al., 2023)	225,534 (38,691)
RISK	General risk tolerance	(Karlsson Linnér et al., 2019)	466,571
NSEX	Lifetime number of sexual partners	(Karlsson Linnér et al., 2019)	370,711
AFB	Age at first birth	(Mills et al., 2021)	542,901
AFS	Age at first sexual intercourse	(Mills et al., 2021)	397,338
Internalizing traits			
DEP	Lifetime diagnosis of major depressive disorder or broad self-reported depression	(Howard et al., 2019)	500,199 (170,756)
NEURO	Weighted neuroticism score based on the response to 12 items	(Nagel, Jansen, et al., 2018)	390,278
WORRY-NEURO	Worry subcluster of NEURO	(Nagel, Jansen, et al., 2018)	348,219
DEP-NEURO	Depressed affect subcluster of NEURO	(Nagel, Jansen, et al., 2018)	357,957
MOOD	Experiencing mood swings	(Nagel, Watanabe, et al., 2018)	373,733 (168,180)

For the three most studied drugs, tobacco, alcohol, and cannabis, we selected at least one SUB and one SUD or SUD-proxy. As tobacco use behavior, we chose ever be a regular smoker (EverSmk) (Saunders et al., 2022), which reflects smoking initiation. To assess tobacco use disorder (TUD), we generated our own GWAS of TUD using UK Biobank data (further information in Supplementary Methods). UK Biobank data was obtained under accession number 27412. We also used Cigsperday (Saunders et al., 2022), which is a good ‘genetic proxy’ of nicotine dependence (Sanchez-Roige et al., 2021). As alcohol use behaviors, we selected DrnkWk (Saunders et al., 2022), a score comprising the items related to alcohol consumption of the Alcohol Use Disorders Identification Test (AUDIT-C) (Sanchez-Roige et al., 2019) and AUDIT-P (Sanchez-Roige et al., 2019). AUD was assessed by an AUD GWAS (Icick et al., 2023). For cannabis, we selected lifetime cannabis use (CANN) (Pasman et al., 2018) and CUD (Johnson et al., 2020).

As externalizing traits, we chose those four traits that were previously used to construct an externalizing genetic factor and do not involve substance use (Karlsson Linnér et al., 2021): ADHD (Demontis et al., 2023), AFS (Mills et al., 2021), number of sexual partners (NSEX) (Karlsson Linnér et al., 2019), and general risk tolerance (RISK) (Karlsson Linnér et al., 2019). In addition, AFB (Mills et al., 2021) and childhood aggressive behavior (AGG) (Ip et al., 2021), related to externalizing behavior (Baselmans et al., 2021; Mills et al., 2021), were also included.

For internalizing traits, different phenotypes of depression and anxiety were selected: a broader diagnosis of depression (DEP) (Howard et al., 2019), MDD (Wray et al., 2018), a continuous trait for anxiety based on the Generalized Anxiety Disorder 2-item scale (GAD) (Levey et al., 2020) and self-reported anxiety (ANX) (http://www.nealelab.is/uk-biobank/). Moreover, neuroticism, a core feature of internalizing behavior (Griffith et al., 2010), measured by a neuroticism score based on 12 items (NEURO) was also included (Nagel, Jansen, et al., 2018). Specific subclusters and items of NEURO were also analyzed: depressed affect subcluster of NEURO (DEP-NEURO) (Nagel, Jansen, et al., 2018), worry subcluster of NEURO (WORRY-NEURO) (Nagel, Jansen, et al., 2018), mood instability (MOOD) (Nagel, Watanabe, Stringer, Posthuma, & Van Der Sluis, 2018), and often feeling lonely (LONE) (Nagel, Watanabe, et al., 2018). The two subclusters of NEURO show distinct patterns of genetic correlation with behavioral, neuropsychiatric, anthropometric, and health-related phenotypes, while a genetic correlation of NEURO is a mix of both subclusters (Nagel, Jansen, et al., 2018; Nagel, Watanabe, et al., 2018). Subjective well-being (SWB) (Kim et al., 2022) was also selected as it is highly correlated with anxious/depressive behavior (Bartels, Cacioppo, Van Beijsterveldt, & Boomsma, 2013). Other aspects of loneliness were also included: the frequency of being able to confide in someone close (CONF) (http://www.nealelab.is/uk-biobank/) and a combined multi-trait GWAS of loneliness and social isolation (LONE-MTAG) (Day, Ong, & Perry, 2018), which is strongly genetically correlated with neuroticism and depressive symptoms.

### Data analysis

We applied MiXeR, version 1.2.0, to characterize the pattern of genetic overlap between SUBs and SUDs and externalizing and internalizing traits using GWAS summary statistics (Frei et al., 2019). First, univariate MiXeR was applied to each trait to estimate their polygenicity, that is the fraction of causal variants in the genome, and discoverability, that is the causal effect size variance, maximizing likelihood estimation (Holland et al., 2020). SNP-based heritability (h^2^_SNP_) is derived from the product of these estimates, taking heterozygosity into account (Frei et al., 2019). Polygenicity is presented as the number of causal variants required to explain 90% of h^2^_SNP_. This threshold of 90% is applied to exclude those variants with infinitesimally small effects. Next, bivariate MiXeR was applied to assess the pattern of genetic overlap between each pair of traits. Using the parameters estimated in univariate MiXeR for each trait, bivariate MiXeR estimates the number of shared causal variants, the number of unique causal variants specific to each trait and the correlation of effect sizes of shared variants (r_gs_). To enhance result interpretation, we assessed the degree of genetic overlap between each pair of traits by quantifying the percentage of shared variants with respect to the SUB/SUD. To assess model fit, the difference between the Akaike information criterion (AIC) for the reference model and the MiXeR-fitted model was calculated. Positive AIC differences indicate that the MiXeR-fitted estimates are more informative than the reference model. The minimum and maximum possible overlap are used as the reference models in bivariate MiXeR. The minimum possible overlap between two traits is restricted by their genome-wide genetic correlation and the maximum possible overlap is restricted by the polygenicity of the least polygenic trait. Further information about MiXeR is provided in Supplementary Methods and the original publication (Frei et al., 2019).

It is well known that results for methods based on summary statistics are dependent on the power of the GWAS. Underpowered GWAS may lead to unreliable results. To limit bivariate MiXeR analyses to the most well-powered GWAS, we selected GWAS according to their univariate MiXeR model fit. Specifically, we selected those traits with substantial improvement in model fit by the causal mixture model in comparison to the infinitesimal model, which assumes all variants as causal. An arbitrary threshold of 50 as the difference in AIC between the infinitesimal model and the causal mixture model was applied. The difference in AIC between these two models is strongly correlated with the power of a GWAS, roughly estimated as the product of the effective sample size and h^2^_SNP_ in our data (Pearson’s correlation coefficient = 0.92, 95% CI: 0.83–0.96) (Supplementary Methods and Supplementary Figure 1). Using BIC as an alternative measure of model fit would lead to a similar selection (Supplementary Table 2). For Cigsperday GWAS, we excluded the cluster of neuronal nicotinic acetylcholine receptor (nAchR) genes at chromosome 15 because its strong effect on this phenotype violates MiXeR’s assumption that the effect sizes of causal variants are normally distributed. Further details about the exclusion of the cluster of nAchR genes at chromosome 15 for Cigsperday are shown in Supplementary Methods.

## Results

**Figure 1.**
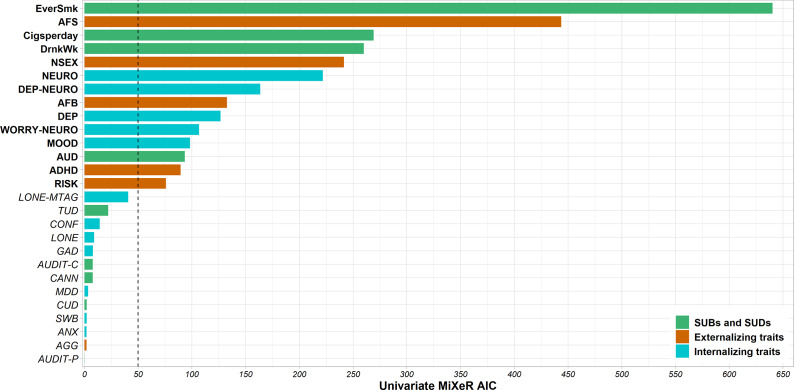
GWAS filtering according to their univariate MiXeR AIC. GWAS selected for MiXeR analyses, that is, those with a difference between the AIC calculated for the infinitesimal model and the AIC calculated for the causal mixture model higher than 50, are highlighted in bold. The dashed line represents univariate MiXeR AIC difference = 50. Traits are described in Table 1 and Supplementary Table 1. ADHD, attention deficit hyperactivity disorder; AFB, age at first birth; AFS, age at first sexual intercourse; AGG, childhood aggressive behavior; ANX, self-response to ever being diagnosed by anxiety, nerves or generalized anxiety disorder; AUD, alcohol use disorder; AUDIT-C, items related to alcohol consumption of the alcohol use disorders identification test; AUDIT-P, items related to problematic consequences of drinking of the alcohol use disorders identification test; CANN, lifetime cannabis use; Cigsperday, average number of cigarettes smoked per day; CONF, frequency of being able to confide in someone close; CUD, cannabis use disorder; DEP, lifetime diagnosis of major depressive disorder or broad self-reported depression; DEP-NEURO, depressed affect subcluster of neuroticsm; DrnkWk, average number of drinks drunk each week; EverSmk, ever been a regular smoker; GAD, generalized anxiety disorder; LONE, often feeling lonely; LONE-MTAG, combined multi-trait GWAS of loneliness and social isolation; MDD, major derepssive disorder; MOOD, experiencing mood swings; NEURO, neuroticism; NSEX, lifetime number of sexual partners; RISK, general risk tolerance; SWB, subjectrive well-being; TUD, tobacco use disorder; WORRY-NEURO, worry subcluster of neuroticism.

After excluding GWAS with poor MiXeR model fit, we included the following tobacco and alcohol use behaviors, respectively: EverSmk and DrnkWk; and a SUD or SUD-proxy for each of these substances: Cigsperday and AUD (Figure 1). We included five externalizing traits: AFS, NSEX, AFB, RISK, and ADHD; and five internalizing traits: NEURO, DEP-NEURO, WORRY-NEURO, DEP, and MOOD.

### Univariate MiXeR results

Univariate MiXeR estimated the polygenicity and discoverability of each of the selected traits (Supplementary Table 2). Among SUBs and SUDs, EverSmk was the most polygenic trait (N = 11.6 K causal variants explaining 90% of the h^2^_SNP_, SE = 0.7 K). DrnkWk and AUD had similar polygenicity (DrnkWk: N = 7.7 K, SE = 0.6 K; AUD: N = 7.7 K, SE = 0.3 K). Cigsperday was the least polygenic trait with 4.3 K (SE = 0.6 K) variants involved. All selected externalizing and internalizing traits were highly polygenic with 9.7 K–12.2 K variants explaining 90% of the h^2^_SNP_, except for ADHD with 7.7 K (SE = 0.4 K) causal variants.

### Bivariate MiXeR results

Bivariate MiXeR was used to estimate the pattern of genetic overlap between each of the four selected SUBs and SUDs and the 10 selected externalizing and internalizing traits. Specifically, the percentage of shared variants with respect to the SUB/SUD and the genetic correlation of shared variants were estimated for each pair of traits. Full results can be found in Supplementary Table 3.

#### Bivariate MiXeR model fit

For almost all bivariate analyses, both AIC compared to minimum and maximum overlap were positive, indicating a good model fit (Supplementary Table 3). Minimum AIC was negative for AUD with NSEX and DEP, indicating that the overlap modeled by MiXeR was not distinguishable from the minimum overlap taking into account the genetic correlation between both traits. Maximum AIC was negative for some analyses for which there was a high overlap between both traits, indicating that the overlap estimated by MiXeR was not distinguishable from complete genetic overlap.

#### Pattern of genetic overlap between externalizing/internalizing traits and SUBs

**Figure 2.**
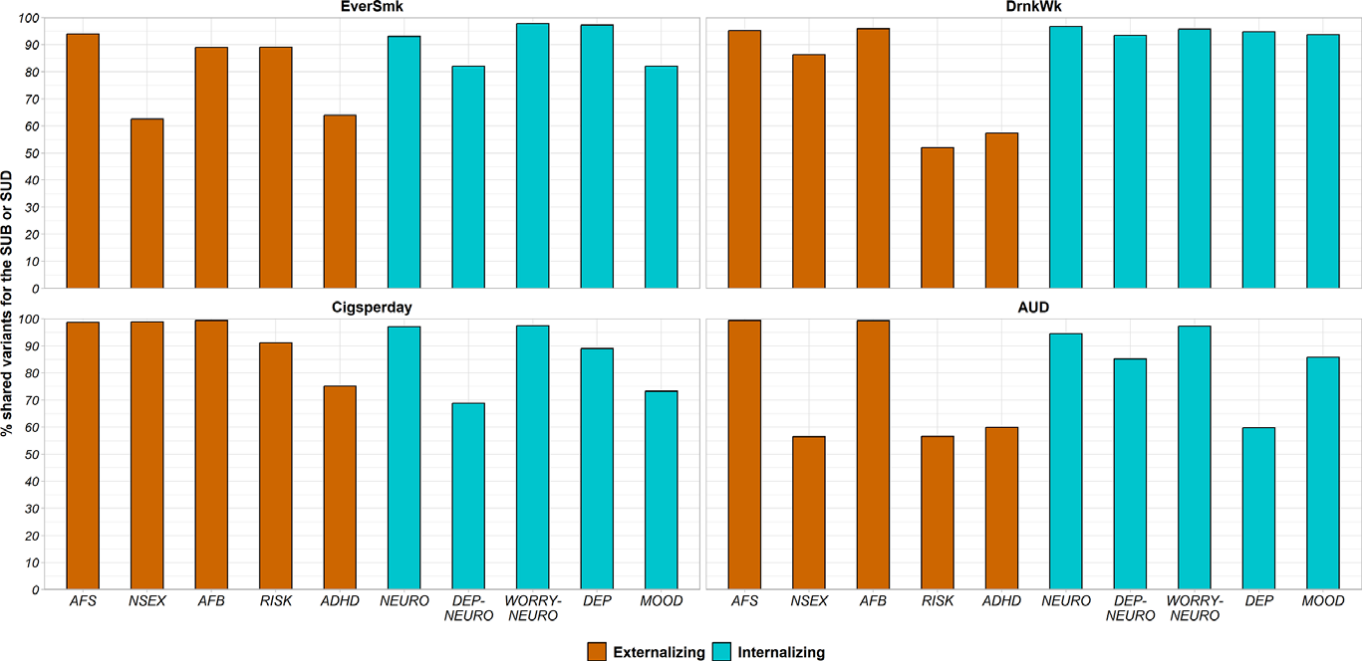
Percentage of variants of each SUB (EverSmk and DrnkWk) or SUD/SUD-proxy (Cigsperday and AUD) shared with each externalizing (colored in orange) or internalizing (colored in green) trait. Traits are described in Table 1. ADHD, attention deficit hyperactivity disorder; AFB, age at first birth; AFS, age at first sexual intercourse; AUD, alcohol use disorder; Cigsperday, average number of cigarettes smoked per day; DEP, lifetime diagnosis of major depressive disorder or broad self-reported depression; DEP-NEURO, depressed affect subcluster of neuroticism; DrnkWk, average number of drinks drunk each week; EverSmk, ever been a regular smoker; MOOD, experiencing mood swings; NEURO, neuroticism; NSEX, lifetime number of sexual partners; RISK, general risk tolerance; WORRY-NEURO, worry subcluster of neuroticism.

**Figure 3.**
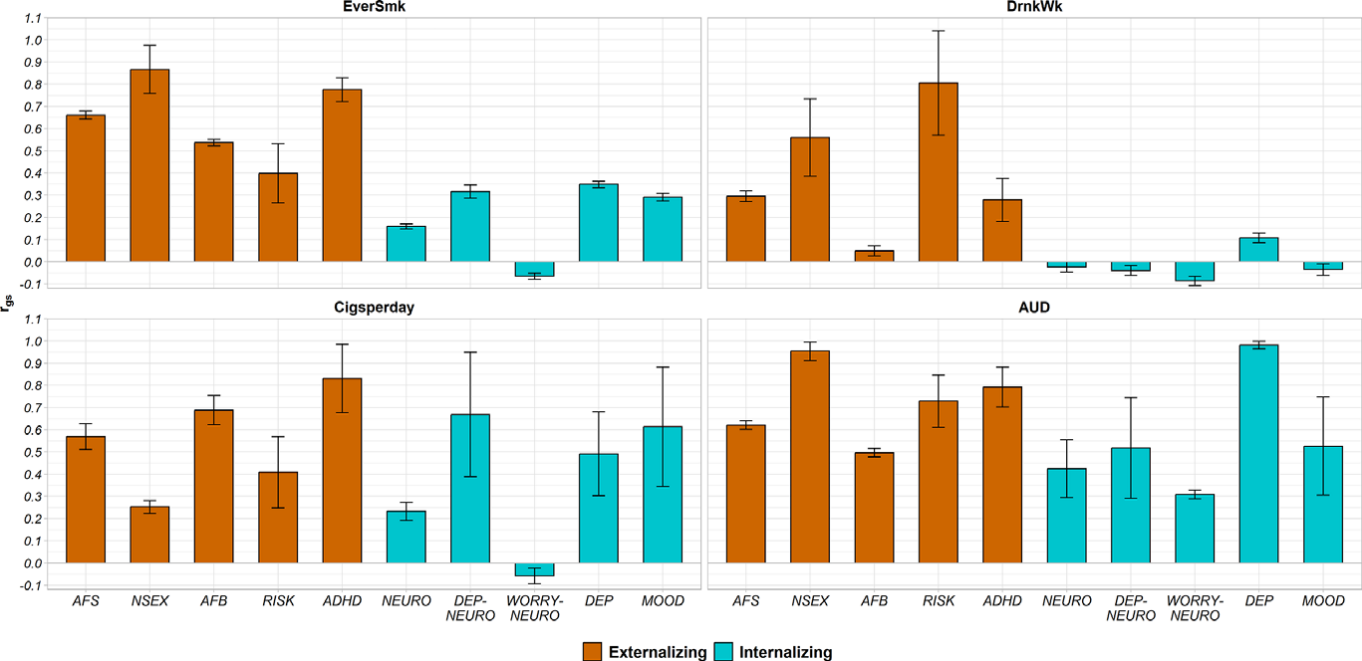
Genetic correlation of the shared variants (r_gs_) between each SUB (EverSmk and DrnkWk) or SUD/SUD-proxy (Cigsperday and AUD) and each externalizing (colored in orange) or internalizing (colored in green) trait. Traits are described in Table 1. Error bars represent ±1 SD. The direction was reversed for AFB and AFS to indicate the genetic correlation with a higher externalizing behavior. ADHD, attention deficit hyperactivity disorder; AFB, age at first birth; AFS, age at first sexual intercourse; AUD, alcohol use disorder; Cigsperday, average number of cigarettes smoked per day; DEP, lifetime diagnosis of major depressive disorder or broad self-reported depression; DEP-NEURO, depressed affect subcluster of neuroticism; DrnkWk, average number of drinks drunk each week; EverSmk, ever been a regular smoker; MOOD, experiencing mood swings; NEURO, neuroticism; NSEX, lifetime number of sexual partners; RISK, general risk tolerance; WORRY-NEURO, worry subcluster of neuroticism.

The percentage of SUB causal variants shared with externalizing traits ranged from 52.0% to 95.9% (Figure 2 and Supplementary Figure 2). The genetic correlation of shared variants was moderate to high with almost all externalizing traits (absolute values of r_gs_ = 0.28–0.87), except between DrnkWk and AFB (absolute value of r_gs_ = 0.05) (Figure 3).

In the case of internalizing traits, genetic overlap with SUBs was high (Figure 2 and Supplementary Figure 2). EverSmk and DrnkWk shared more than 82% and 93% of their causal variants with internalizing traits, respectively (Figure 2). The genetic correlation of shared variants with internalizing traits was clearly lower than with externalizing traits (absolute values of r_gs_ = 0.02–0.35) (Figure 3).

Despite following the same general pattern of genetic overlap, some differences emerged between tobacco use behavior (EverSmk) and alcohol use behavior (DrnkWk). EverSmk exhibited a slightly higher genetic correlation of shared variants (absolute values of r_gs_ = 0.40–0.87) with externalizing traits compared to DrnkWk (absolute values of r_gs_ = 0.05–0.81) (Figure 3). Similarly, internalizing traits exhibited a higher genetic correlation of shared variants with EverSmk (absolute values of r_gs_ = 0.06–0.35) compared to DrnkWk (absolute values of r_gs_ = 0.02–0.11). Notably, DrnkWk showed a negative genetic correlation of shared variants, although close to 0, with all internalizing traits except for DEP, while EverSmk only displayed a negative genetic correlation of shared variants with WORRY-NEURO among internalizing traits.

#### Pattern of genetic overlap between externalizing/internalizing traits and SUDs/SUD-proxies

The percentage of causal variants for SUDs and SUD-proxies shared with externalizing traits ranged from 56.5% to 99.4% (Figure 2 and Supplementary Figure 2). The genetic correlation of shared variants for SUDs and SUD-proxies with externalizing traits was relatively high (absolute values of r_gs_ = 0.25–0.95) (Figure 3). In all pairs, a higher value for externalizing behavior was positively correlated with both SUBs and SUDs among the shared variants (Figure 3).

Genetic overlap with internalizing traits for SUDs and SUD-proxies (59.7%–97.4%) was more variable than for SUBs (82.0%–97.8%) (Figure 2 and Supplementary Figure 2). Cigsperday and AUD demonstrated a higher genetic correlation of shared variants with internalizing traits, except WORRY-NEURO, (absolute values of r_gs_ = 0.23–0.98) than SUBs (absolute values of r_gs_ = 0.02–0.35) (Figure 3). In contrast to the remaining internalizing traits, WORRY-NEURO showed a similar pattern of genetic overlap with both SUBs and SUDs. WORRY-NEURO shared a large number of variants (>95%) with all SUBs and SUDs, despite possessing low genetic correlation of shared variants (Figure 2 and Figure 3). The direction of the genetic correlation of shared variants was positive for pairs involving AUD and internalizing traits, contrary to DrnkWk (Figure 3). By contrast, the direction of the genetic correlation of shared variants with internalizing traits was positive for both tobacco traits.

#### Relationship between the genetic correlation of shared variants and the number of shared causal variants

**Figure 4.**
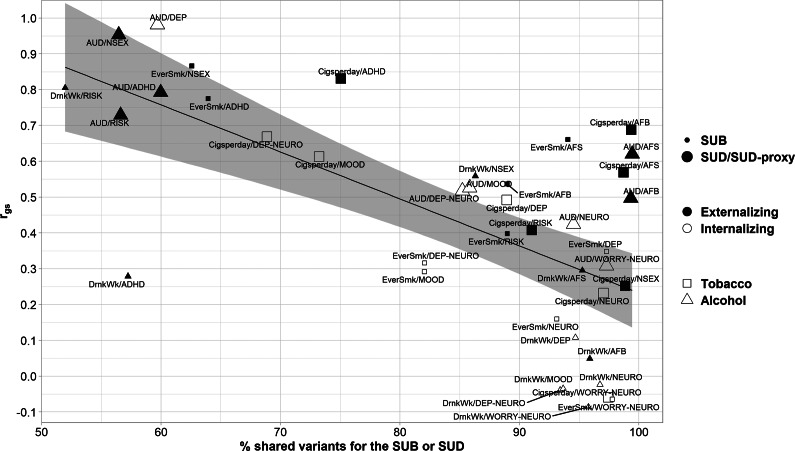
Scatter plot illustrating the negative relationship between the genetic correlation of shared variants (r_gs_) and the percentage of shared variants across analyzed trait pairs. The solid line represents the linear regression, while the shaded region depicts the 95% confidence interval. On the x-axis, the percentage of shared variants between each SUB or SUD and each externalizing or internalizing trait with respect to the SUB/SUD is displayed. The y-axis shows the genetic correlation of shared variants between each SUB or SUD and each externalizing or internalizing trait. For pairs involving AFB and AFS, the direction of the genetic correlation of shared variants was reversed to indicate the genetic correlation with a higher externalizing behavior. Pairs involving SUBs (EverSmk and DrnkWk) are denoted by smaller dots, while pairs involving SUDs or SUD-proxies (Cigsperday and AUD) are represented by larger dots. Pairs involving externalizing or internalizing traits are colored in black or white, respectively. Pairs involving tobacco use behaviors or disorders (EverSmk and Cigsperday) are depicted by squares and pairs involving alcohol use behaviors or disorders (DrnkWk and AUD) are represented by triangles. ADHD, attention deficit hyperactivity disorder; AFB, age at first birth; AFS, age at first sexual intercourse; AUD, alcohol use disorder; Cigsperday, average number of cigarettes smoked per day; DEP, lifetime diagnosis of major depressive disorder or broad self-reported depression; DEP-NEURO, depressed affect subcluster of neuroticism; DrnkWk, average number of drinks drunk each week; EverSmk, ever been a regular smoker; MOOD, experiencing mood swings; NEURO, neuroticism; NSEX, lifetime number of sexual partners; RISK, general risk tolerance; SUB, substance use behaviors; SUD, substance use disorders, WORRY-NEURO, worry subcluster of neuroticism.

An inverse relationship was observed between the genetic correlation of shared variants and the number of shared causal variants for each pair of traits, with a significant negative correlation between these variables (r = −0.66, P = 4.32x10^−6^) (Figure 4). Those pairs of traits exhibiting a lower genetic correlation of shared variants than expected given the number of shared variants comprised predominantly SUBs and internalizing traits (small open triangles and squares in Figure 4). In contrast, pairs showing a genetic correlation of shared variants higher than expected based on the number of shared variants involved SUDs or SUD-proxies with externalizing traits (large filled triangles and squares in Figure 4).

Among the analyzed trait pairs, a subgroup exhibited a comparable degree of genetic overlap (>90% of shared causal variants) (Figure 4). Some pairs involving SUDs and SUD-proxies with externalizing traits demonstrated a relatively high genetic correlation of shared variants (absolute values of r_gs_ = 0.50–0.69), despite their high degree of genetic overlap, such as Cigsperday and AFB or AUD and AFS. Conversely, pairs with a similar degree of genetic overlap comprising SUDs or SUD-proxies with internalizing traits demonstrated a lower genetic correlation of shared variants (absolute values of r_gs_ = 0.06–0.42), such as Cigsperday and WORRY-NEURO or Cigsperday and NEURO. However, it was much lower for SUBs, notably DrnkWk, and internalizing traits (absolute values of r_gs_ = 0.02–0.16, except for EverSmk and DEP (absolute value of r_gs_ = 0.35)).

## Discussion

Using MiXeR, we detected a distinct pattern of genetic overlap between SUBs and SUDs with externalizing and internalizing traits. SUBs (i.e. EverSmk and DrnkWk) and internalizing traits demonstrated a notably high genetic overlap but a particularly low genetic correlation of shared variants. This finding may be explained by a divergence in the effect distribution of the numerous shared risk variants between SUBs and internalizing traits, suggesting a limited role in the genetics of internalizing problems at SUBs. By contrast, SUBs showed a higher genetic correlation of shared variants with externalizing traits, suggesting that externalizing problems may have a stronger genetic influence on SUBs, albeit with variations depending on the substance. With regards to SUDs or SUD-proxies (i.e. Cigsperday and AUD), we observed a notably high genetic correlation of shared variants with externalizing traits, also in cases of high genetic overlap. This observation suggests that most risk variants for SUDs and SUD-proxies are shared with externalizing problems, and these variants show a similar effect distribution on both traits. A similar pattern, although not as marked, was found for pairs involving SUDs and SUD-proxies with internalizing problems. These findings suggest that while both externalizing and internalizing pathways may be involved in SUDs, externalizing pathway seems to play a more consistent role.

Bivariate MiXeR revealed a substantial genetic overlap between SUBs and SUDs and both externalizing and internalizing traits. A similar pattern was already identified in a previous study from our group between mental disorders and other traits such as intelligence, educational attainment, neuroticism, and SWB (Hindley et al., 2022). This study concluded that a large number of pleiotropic variants are involved in all these traits with differences in the effect size distribution of these pleiotropic variants playing a more prominent role in the genetic predisposition for a given trait than trait-specific genetic variants. Considering our results, this conclusion can also be extended to the genetic overlap of externalizing and internalizing traits with SUBs and SUDs. Our study identified two distinct patterns within this general framework. SUBs and internalizing traits showed a high genetic overlap but a genetic correlation of shared variants close to zero, suggesting a distinctly divergent effect distribution within both traits. Conversely, certain pairs involving SUDs or SUD-proxies with externalizing traits displayed a similar distribution of effects as well as a high genetic overlap, suggesting a shared genetic basis among these traits.

Our results are consistent with previous studies suggesting that, while the internalizing pathway plays a limited role at the initial stages of substance consumption, it has a stronger impact at more severe levels of use (Hussong et al., 2011; Ning et al., 2020). A different genetic relationship of internalizing traits with SUBs and SUDs has already been suggested using MiXeR (Icick et al., 2022). In this previous study, mood instability showed a different pattern of genetic overlap with alcohol consumption, defined as AUDIT-C, and AUD. Here, we have expanded on these findings by using several internalizing traits in addition to mood instability and a different alcohol consumption trait (DrnkWk). Our findings are also consistent with previous studies suggesting a role of the externalizing pathway at all substance use stages. An effect of the externalizing pathway on substance use initiation was suggested in a previous study which found that the shared genetic susceptibility between schizophrenia and smoking initiation is related to externalizing behavior (Al-Soufi & Costas, 2023). Importantly, our results emphasize the substantial role of the externalizing pathway in SUDs, consistent with previous studies supporting a clearer association of externalizing problems with severe substance use compared to substance consumption and internalizing problems (Edwards, Gardner, Hickman, & Kendler, 2016; Ning et al., 2020). Previous studies have implicated the genetic susceptibility to both externalizing and internalizing problems in SUDs. A recent study found that an addiction risk factor based on problematic alcohol use, problematic tobacco use, CUD, and opioid use disorder GWAS correlated genetically with externalizing behavior (absolute r_g_ = 0.35–0.52), as well as with substance use to cope with anxiety (r_g_ = 0.63) and depression (r_g_ = 0.60), suggesting a strong association of externalizing and internalizing behavior with SUDs (Hatoum et al., 2023). Another study based on a similar addiction risk factor found that the association between this addiction risk factor and risk-taking significantly decreased after taking substance use into account (Hatoum et al., 2021). In contrast, genetic susceptibility to substance use had a small effect on the association between this addiction risk factor and neuroticism. Our findings are compatible with these results suggesting that, in contrast to the internalizing pathway, the effect of the externalizing pathway on SUDs may be partially mediated by substance use.

For SUDs and SUD-proxies, both tobacco and alcohol showed comparable patterns of genetic overlap with externalizing and internalizing traits. However, differences emerged between the tobacco (EverSmk) and alcohol (DrnkWk) use behaviors. EverSmk displayed a higher genetic correlation of shared variants with externalizing and internalizing traits compared to DrnkWk. Moreover, the genetic correlation of shared variants of internalizing traits with DrnkWk was close to 0, suggesting that the internalizing pathway may have a minimal effect on alcohol use. In contrast to tobacco use, DrnkWk is a highly heterogenous trait, combining the frequency of use and quantity per drinking occasion (Mallard et al., 2022; Sanchez-Roige et al., 2021). These dimensions show opposite genetic correlations with traits related to socioeconomic status, psychiatric disorders, and psychological traits (Marees et al., 2019). As a consequence of this heterogeneity, DrnkWk is expected to be less influenced by the internalizing and externalizing pathways than use of tobacco or other substances.

The present study has some limitations. First, we cannot conclude that there is a causal effect of externalizing and internalizing problems on SUBs and SUDs on the basis of MiXeR results alone. We can only assess whether there is a shared genetic susceptibility between both traits and its effect distribution on both traits. Second, some of the MiXeR models were not distinguishable from a complete genetic overlap or the minimum genetic overlap allowed for a given genetic correlation. Therefore, larger GWAS based on a more precise phenotype definition is required to achieve more accurate estimates of the number of shared and unique genetic variants. Third, a lack of well-powered GWAS for other substances only allowed the analysis of substance use for two legal substances: alcohol and tobacco. Thus, our conclusions may not generalize to other substances. Fourth, phenotype definition remains a major topic in psychiatric genetics. A continuous trait for maladaptive alcohol use would assess more accurately the level of substance dependence of an individual. Adding data regarding the course of SUD (age at onset, continuous versus discontinuous use, duration of SUD, speed, and age of transition between use and SUD) may be relevant in refining the genetic component of SUD. This limitation of the dichotomous approach is extensive to other traits used in this study. Furthermore, differences in the procedures used to ascertain cases may be associated with measurement bias. In general, there is a need for more comprehensive assessments of GWAS samples regarding traits of relevance in psychiatry. Unfortunately, most of the phenotypic refinements susceptible to improved transfer to biology or the clinics occur at the expense of sample size, thus reducing statistical power. Fifth, there is considerable comorbidity between externalizing and internalizing behavior, which makes it difficult to distinguish the separate effects of each analyzed pathway. Moreover, many GWAS rely on samples like UK Biobank, which may not accurately represent the general population in terms of gender, socio-economic status, or ancestry, potentially biasing our results (Fry et al., 2017). In addition, since MiXeR assumes that the effect size of causal variants is normally distributed, the cluster of nAchR genes at chromosome 15 was excluded from our Cigsperday GWAS. MiXeR also assumes an additive model of genetic effects, while gene–environment interactions, epistasis, and dominance effects may play an important role, particularly in personality traits such as neuroticism (Boomsma et al., 2018; Keller, Coventry, Heath, & Martin, 2005). Violation of the MiXeR’s assumption of additivity may therefore contribute to the weak genetic correlations with neuroticism. Furthermore, we did not provide statistical significance for our results, as formal significance tests based on MiXeR results are not feasible. Finally, although we used several sexual behaviors as representative traits of genetic susceptibility for externalizing behavior, these complex phenotypes are affected by gene–environment correlations attributable to social, economic, and political processes (Abdellaoui, Dolan, Verweij, & Nivard, 2022). Therefore, a cautionary remainder is needed to avoid misinterpretation of genomic findings that may lead to stigmatization.

In summary, we found a different genetic relationship between SUBs and SUDs with externalizing and internalizing traits, suggesting a larger involvement of the externalizing and internalizing pathways in severe substance use. In addition, the externalizing pathway seems to play a more prominent role throughout the progression of substance use than the internalizing pathway. These findings improve our understanding of the origins of substance use and SUDs, which may inform attempts to advance prevention and treatment strategies.

## Supporting information

Al-Soufi et al. supplementary material Al-Soufi et al. supplementary material

## Supplementary material

The supplementary material for this article can be found at http://doi.org/10.1017/S0033291725000108.
