# Supplementary material for: Polygenic overlap of substance use behaviors and disorders with externalizing and internalizing problems independent of genetic correlations
Source: Psychol Med. 2025 Mar 31;55:e100. doi: 10.1017/S0033291725000108 (PMC12094651; doi:10.1017/S0033291725000108)
Supplement: Al-Soufi et al. supplementary material [file S0033291725000108sup001.docx]

**SUPPLEMENTARY INFORMATION**

**Polygenic overlap of substance use behaviors and disorders with externalizing and internalizing problems independent of genetic correlations**

Laila Al-Soufi, Guy Hindley, Linn Rødevand, Alexey A. Shadrin, Piotr Jaholkowski, Vera Fominykh, Romain Icick, Markos Tesfaye, Javier Costas and Ole A. Andreassen

**SUPPLEMENTARY METHODS**

**Tobacco use disorder GWAS**

We performed a TUD GWAS using UK Biobank data. UK Biobank data was obtained under accession number 27412. Participants with a diagnosis of a mental and behavioral disorder due to the use of tobacco with either harmful use or a dependence syndrome (as coded in ICD10 F17) were cases (N=19,085). Participants without a mental and behavioral disorder due to the use of tobacco were used as controls (N=390,450). Genotyping, imputation, and central quality control procedures for the UK Biobank genotypes are described in detail elsewhere (Bycroft et al., 2018). The analysis was restricted to individuals who self-identified as "White British" and had very similar genetic ancestry based on a principal components analysis (as defined by UK Biobank data field 22006). Sex, age and 20 genetic PCs were included as covariates for GWAS analysis. Genetic variants with an INFO score < 0.8 and minor allele count < 20 were excluded from the GWAS leaving 19,972,520 variants.

**MiXeR analysis**

We applied MiXeR to estimate the number of causal variants for each trait and the number of unique and shared causal variants between each pair of traits (Frei et al., 2019). MiXeR makes use of Gaussian mixture model to infer the genetic architecture of traits. Under this model, MiXeR assumes that for a complex trait there is a mixture of causal variants, i.e., causal variants with normally distributed additive genetic effects on the trait, and noncausal variants with zero effect. In the cross-trait analysis, MiXeR models additive genetic effects as a mixture of four components: shared causal variants, unique causal variants specific to each trait and noncausal variants.

Model fit is assessed by the difference between the Akaike information criterion (AIC) for the MiXeR-fitted model and a reference model. Positive AIC differences indicate that the MiXeR-fitted estimates are more informative than the reference model. For univariate MiXeR, an infinitesimal model assuming all variants as causal is used as reference. For bivariate MiXeR, the minimum and maximum possible overlap are used as the reference models. The minimum possible overlap between two traits is restricted by their genome-wide genetic correlation and the maximum possible overlap is restricted by the polygenicity of the least polygenic trait.

SNPs within the major histocompatibility complex (chr6:26000000-34000000) were excluded, owing to its complex LD structure. The 1000 Genomes phase 3 European samples were used as the reference panel to calculate linkage disequilibrium information. Twenty sets of approximately 2 million SNPs with MAF>0.05 and r^2^<0.8 were randomly generated from reference panel. Each MiXeR analysis comprised 20 iterations, with each iteration analyzing one of these sets of SNPs. Mean estimates and SDs of polygenicity, discoverability, genetic overlap and genetic correlation within the shared component were computed from each set of 20 iterations.

Effective sample size (computed as 4/(1/N cases+1/N controls)) was used as sample size in MiXeR for case-control GWAS. For those GWAS with a highly variable sample size depending on the SNP, i.e., alcohol use disorder (AUD) GWAS (Icick et al., 2023) and major depressive disorder (MDD) GWAS (Wray et al., 2018), SNP-specific effective sample sizes were used.

**Exclusion of nicotinic receptor genes cluster at chromosome 15 for Cigsperday MiXeR analyses**

The cluster of neuronal nicotinic acetylcholine receptor (nAchR) genes at chromosome 15, i.e., *CHRNA5/CHRNA3/CHRNB4*, has a very strong effect on Cigsperday GWAS. A high variance in the effect of the causal variants, with genes having a much larger effect than other genes on the trait, violates MiXeR assumption of a unique distribution of causal variants. So, we excluded *CHRNA5/CHRNA3/CHRNB4* from Cigsperday GWAS summary statistics for MiXeR analysis. Specifically, we excluded ±500 kb region around *CHRNA5/CHRNA3/CHRNB4*. A comparison of univariate MiXeR results with Cigsperday without excluding *CHRNA5/CHRNA3/CHRNB4* is shown in Supplementary Table 4. We also excluded this cluster from those selected externalizing and internalizing traits for bivariate MiXeR analyses with Cigsperday.

**Correlation between power of a GWAS and univariate MiXeR AIC**

Power of a GWAS was roughly estimated as the product of the effective sample size (N_eff_) and the SNP-based heritability (h^2^_SNP_), as in Hindley et al. (2022). Linkage disequilibrium score regression (LDSC), as implemented in genomicSEM (Grotzinger et al., 2019), was used to estimate observed h^2^_SNP_ from GWAS summary statistics (Bulik-Sullivan et al., 2015). Linkage Disequilibrium (LD) Scores files based on The 1000 Genomes phase 3 European samples were used and GWAS summary statistics were filtered to HapMap3 SNPs. Summary statistic filters were set as default. In case of AUD and MDD, which present considerable differences in N_eff_ per SNP, the 25th percentile was used as N_eff_. Linear regression analysis was used to estimate Pearson's correlation coefficient between GWAS power and univariate MiXeR AIC (Supplementary Figure 1).

**REFERENCES**

Bulik-Sullivan, B., Finucane, H. K., Anttila, V., Gusev, A., Day, F. R., Loh, P.-R., … Neale, B. M. (2015). An atlas of genetic correlations across human diseases and traits. Nature Genetics, 47(11), 1236–1241. doi: 10.1038/ng.3406

Bycroft, C., Freeman, C., Petkova, D., Band, G., Elliott, L. T., Sharp, K., … Marchini, J. (2018). The UK Biobank resource with deep phenotyping and genomic data. *Nature*, *562*(7726), 203–209. doi: 10.1038/s41586-018-0579-z

Day, F. R., Ong, K. K., & Perry, J. R. B. (2018). Elucidating the genetic basis of social interaction and isolation. *Nature Communications*, *9*(1), 1–6. doi: 10.1038/s41467-018-04930-1

Frei, O., Holland, D., Smeland, O. B., Shadrin, A. A., Fan, C. C., Maeland, S., … Dale, A. M. (2019). Bivariate causal mixture model quantifies polygenic overlap between complex traits beyond genetic correlation. *Nature Communications*, *10*(1), 1–11. doi: 10.1038/s41467-019-10310-0

Grotzinger, A. D., Rhemtulla, M., de Vlaming, R., Ritchie, S. J., Mallard, T. T., Hill, W. D., … Tucker-Drob, E. M. (2019). Genomic structural equation modelling provides insights into the multivariate genetic architecture of complex traits. *Nature Human Behaviour*, *3*(5), 513–525. doi: 10.1038/s41562-019-0566-x

Hindley, G., Frei, O., Shadrin, A. A., Cheng, W., O’Connell, K. S., Icick, R., … Andreassen, O. A. (2022). Charting the Landscape of Genetic Overlap Between Mental Disorders and Related Traits Beyond Genetic Correlation. *American Journal of Psychiatry*, *179*(11), 833–843. doi: 10.1176/appi.ajp.21101051

Icick, R., Shadrin, A., Holen, B., Karadag, N., Parker, N., O’Connell, K. S., … Andreassen, O. A. (2023). *Identification of Novel Loci and Cross-Disorder Pleiotropy Through Multi- Ancestry Genome-Wide Analysis of Alcohol Use Disorder in Over One Million Individuals. PREPRINT (Version 1) available at Research Square.* doi: 10.21203/rs.3.rs-3755915/v1

Ip, H. F., van der Laan, C. M., Krapohl, E. M. L., Brikell, I., Sánchez-Mora, C., Nolte, I. M., … Boomsma, D. I. (2021). Genetic association study of childhood aggression across raters, instruments, and age. *Translational Psychiatry*, *11*(1). doi: 10.1038/s41398-021-01480-x

Johnson, E. C., Demontis, D., Thorgeirsson, T. E., Walters, R. K., Polimanti, R., Hatoum, A. S., … Børglum, A. D. (2020). A large-scale genome-wide association study meta-analysis of cannabis use disorder. *The Lancet Psychiatry*, *7*(12), 1032–1045. doi: 10.1016/S2215-0366(20)30339-4

Kim, S., Kim, K., Hwang, M. Y., Ko, H., Jung, S. H., Shim, I., … Won, H. H. (2022). Shared genetic architectures of subjective well-being in East Asian and European ancestry populations. *Nature Human Behaviour*, *6*(7), 1014–1026. doi: 10.1038/s41562-022-01343-5

Levey, D. F., Gelernter, J., Polimanti, R., Zhou, H., Cheng, Z., Aslan, M., … Stein, M. B. (2020). Reproducible Genetic Risk Loci for Anxiety: Results From ∼200,000 Participants in the Million Veteran Program. *American Journal of Psychiatry*, *177*(3), 223–232. doi: 10.1176/appi.ajp.2019.19030256

Nagel, M., Watanabe, K., Stringer, S., Posthuma, D., & Van Der Sluis, S. (2018). Item-level analyses reveal genetic heterogeneity in neuroticism. *Nature Communications*, *9*(1). doi: 10.1038/s41467-018-03242-8

Pasman, J. A., Verweij, K. J. H., Gerring, Z., Stringer, S., Sanchez-Roige, S., Treur, J. L., … Vink, J. M. (2018). GWAS of lifetime cannabis use reveals new risk loci, genetic overlap with psychiatric traits, and a causal effect of schizophrenia liability. *Nature Neuroscience*, *21*(9), 1161–1170. doi: 10.1038/s41593-018-0206-1

Sanchez-Roige, S., Palmer, A. A., Fontanillas, P., Elson, S. L., Adams, M. J., Howard, D. M., … Wilson, C. H. (2019). Genome-wide association study meta-analysis of the alcohol use disorders identification test (AUDIT) in two population-based cohorts. *American Journal of Psychiatry*, *176*(2), 107–118. doi: 10.1176/appi.ajp.2018.18040369

Wray, N. R., Ripke, S., Mattheisen, M., Trzaskowski, M., Byrne, E. M., Abdellaoui, A., … Sullivan, P. F. (2018). Genome-wide association analyses identify 44 risk variants and refine the genetic architecture of major depression. *Nature Genetics*, *50*(5), 668–681. doi: 10.1038/s41588-018-0090-3

Supplementary Figure 1.Scatter plot of univariate MiXeR AIC versus GWAS power, estimated as the product of the effective sample size (N_eff_) and the SNP-based heritability (h^2^_SNP_). Traits are described in Supplementary Table 1.


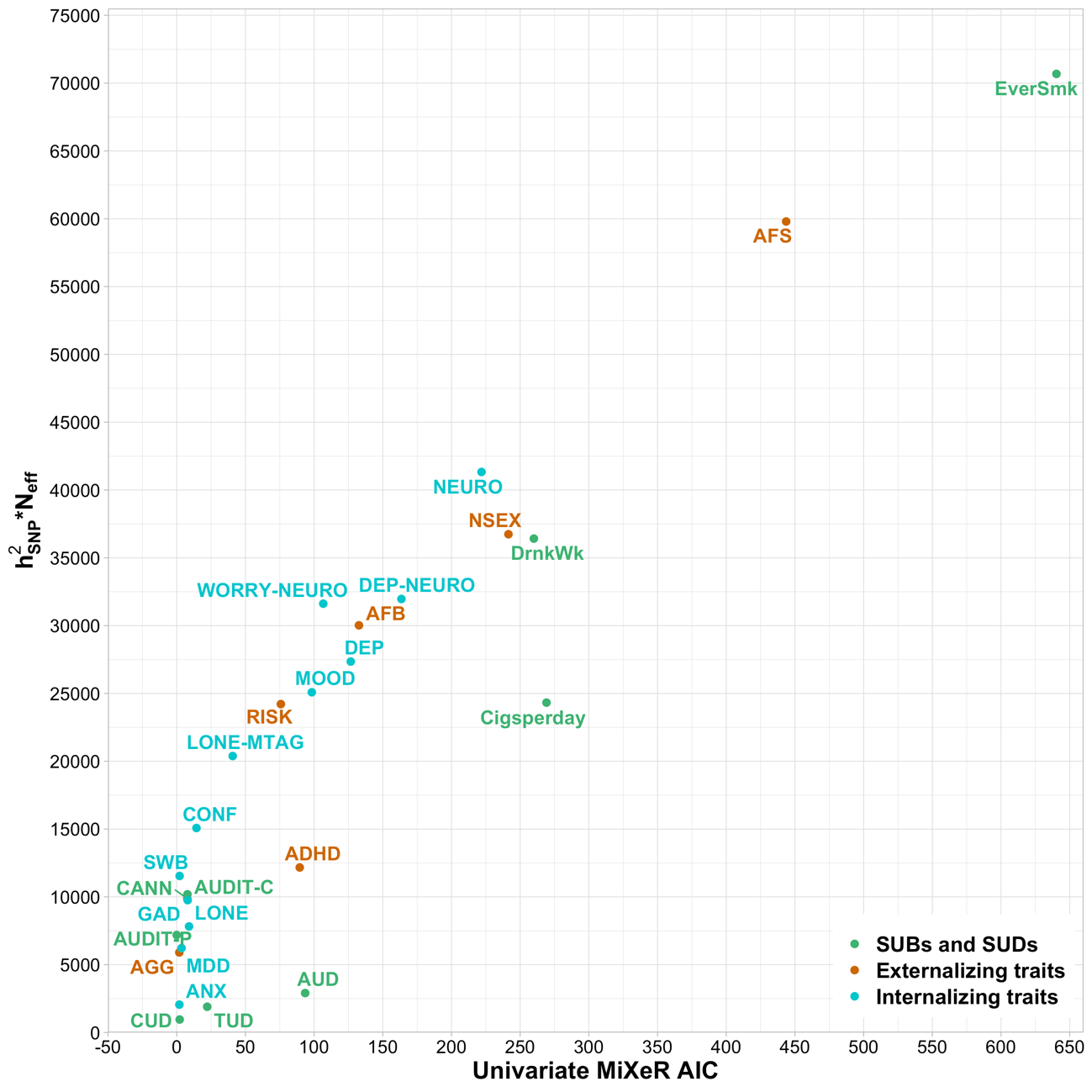


Supplementary Figure 2. MiXeR Venn diagrams showing the number (in thousands) of shared and unique causal variants for each pair of traits. Genome-wide genetic correlation (r_g_) is also shown for each pair of traits.


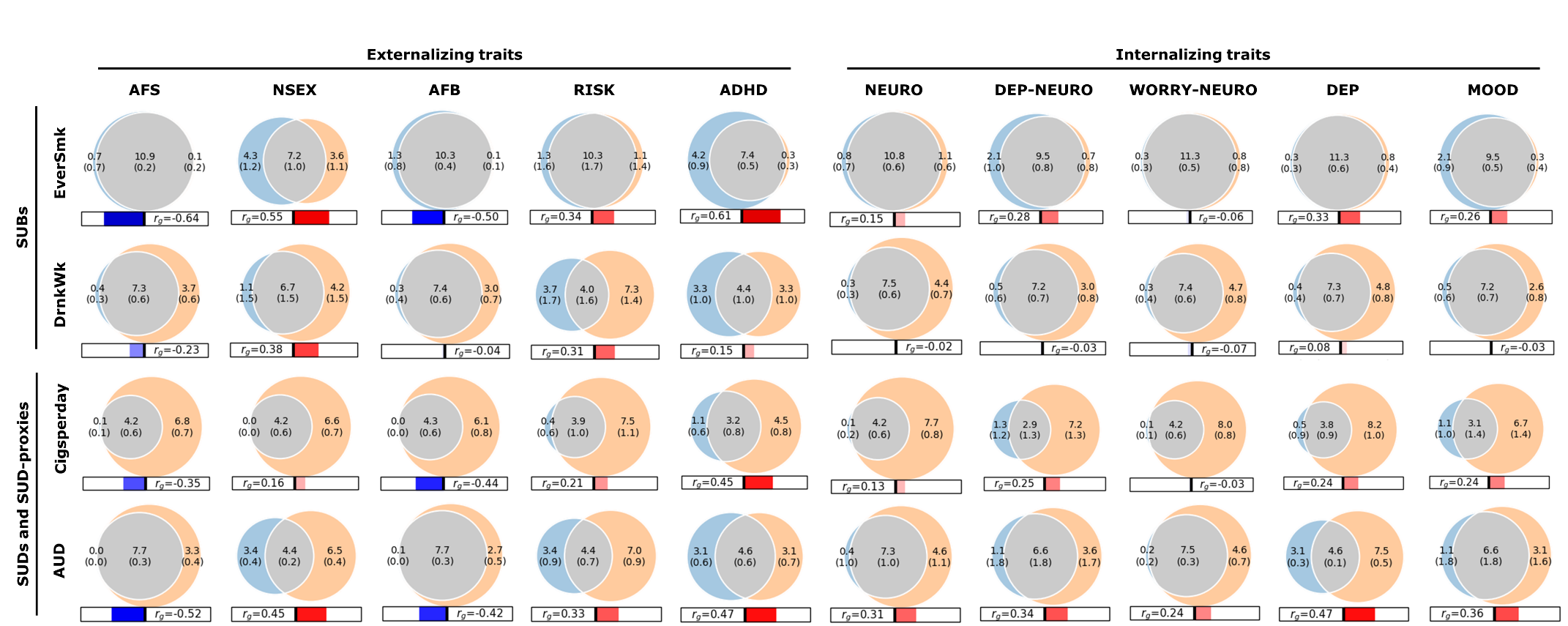


Supplementary Table 1. Additional GWAS summary statistics with poor MiXeR model fit discarded for MiXeR analysis.

| Trait | Description | Source | Sample size (N cases) |
| --- | --- | --- | --- |
| SUBs and SUDs or SUD-proxies | | | |
| TUD | Tobacco use disorder, i.e., harmful use or dependence | Own GWAS using UK Biobank samples | 409,535 (19,085) |
| AUDIT-C | Alcohol Use Disorders  Identification Test (AUDIT): items related to alcohol consumption | (Sanchez-Roige et al., 2019) | 121,604 |
| AUDIT-P | Alcohol Use Disorders Identification Test (AUDIT): items related to problematic consequences of drinking | (Sanchez-Roige et al., 2019) | 121,604 |
| CANN | Lifetime cannabis use, i.e., ever versus never used cannabis | (Pasman et al., 2018) | 162,082 (43,380) |
| CUD | Cannabis use disorder, i.e., cannabis abuse or dependence | (Johnson et al., 2020) | 357,806 (14,080) |
| Externalizing traits | | | |
| AGG | Childhood aggressive behavior assessed on continuous scales | (Ip et al., 2021) | 151,741^1^ |
| Internalizing traits | | | |
| MDD | Major depressive disorder | (Wray et al., 2018) | 173,005 (59,851) |
| GAD | Self-response to Generalized Anxiety Disorder 2-item scale | (Levey et al., 2020) | 175,163 |
| ANX | Self-response to ever being diagnosed by anxiety, nerves or generalized anxiety disorder | Neale lab^b^ | 117,751 (16,730) |
| SWB | Subjective well-being | (Kim et al., 2022) | 311,126 |
| LONE-MTAG | Combined multi-trait GWAS of loneliness and social isolation | (Day, Ong, & Perry, 2018) | 445,024 |
| LONE | Often feeling lonely | (Nagel, Watanabe, Stringer, Posthuma, & Van Der Sluis, 2018) | 376,352 (67,743) |
| CONF | Frequency of being able to confide in someone close | Neale lab^2^ | 350,618 |

^1^: from the original paper.

^2^: http://www.nealelab.is/uk-biobank/

Supplementary Table 2. Univariate MiXeR results.

| Trait | Polygenicity (SD) | Discoverability (SD) | N causal variants (SD)^1^ | AIC^2^ | BIC^2^ |
| --- | --- | --- | --- | --- | --- |
| EverSmk | 3.63x10^-3^ (2.17x10^-4^) | 1.18x10^-5^ (6.50x10^-7^) | 11568 (691) | 640.36 | 649.78 |
| AFS | 3.45x10^-3^ (6.58x10^-5^) | 2.16x10^-5^ (3.76x10^-7^) | 11003 (210) | 443.60 | 454.51 |
| Cigsperday | 1.34x10^-3^ (1.84x10^-4^) | 2.44x10^-5^ (2.93x10^-6^) | 4281 (587) | 269.15 | 278.81 |
| DrnkWk | 2.42x10^-3^ (1.91x10^-4^) | 1.07x10^-5^ (7.06x10^-7^) | 7708 (609) | 259.96 | 269.39 |
| NSEX | 3.39x10^-3^ (1.15x10^-4^) | 1.43x10^-5^ (4.18x10^-7^) | 10814 (368) | 241.46 | 251.31 |
| NEURO | 3.72x10^-3^ (1.44x10^-4^) | 1.33x10^-5^ (5.00x10^-7^) | 11859 (459) | 221.85 | 231.59 |
| DEP-NEURO | 3.19x10^-3^ (1.14x10^-4^) | 1.26x10^-5^ (4.01x10^-7^) | 10170 (364) | 163.54 | 173.27 |
| AFB | 3.26x10^-3^ (1.33x10^-4^) | 8.20x10^-6^ (3.21x10^-7^) | 10402 (423) | 132.62 | 143.45 |
| DEP | 3.79x10^-3^ (1.69x10^-4^) | 8.25x10^-6^ (3.10x10^-7^) | 12083 (539) | 126.69 | 136.47 |
| WORRY-NEURO | 3.80x10^-3^ (1.77x10^-4^) | 1.07x10^-5^ (4.08x10^-7^) | 12123 (564) | 106.65 | 116.38 |
| AUD | 2.42x10^-3^ (1.08x10^-4^) | 2.04 x10^-5^ (8.76x10^-7^) | 7726 (346) | 98.32 | 108.07 |
| MOOD | 3.07x10^-3^ (1.42x10^-4^) | 9.71x10^-6^ (4.23x10^-7^) | 9776 (454) | 93.47 | 104.35 |
| ADHD | 2.41x10^-3^ (1.14x10^-4^) | 2.99x10^-5^ (1.27x10^-6^) | 7696 (363) | 89.52 | 99.15 |
| RISK | 3.56x10^-3^ (1.70x10^-4^) | 6.83x10^-6^ (3.31x10^-7^) | 11353 (541) | 75.79 | 85.65 |
| LONE-MTAG | 3.40x10^-3^ (1.98x10^-4^) | 5.61x10^-6^ (2.88x10^-7^) | 10841 (632) | 40.74 | 50.62 |
| TUD | 1.99x10^-3^ (1.40x10^-4^) | 3.11x10^-5^ (1.91x10^-6^) | 6351 (445) | 22.20 | 32.08 |
| CONF | 4.12x10^-3^ (3.26x10^-4^) | 4.57x10^-6^ (3.43x10^-7^) | 13139 (1038) | 14.35 | 24.22 |
| LONE | 4.98x10^-3^ (4.87x10^-4^) | 5.16x10^-6^ (4.17x10^-7^) | 15867 (1553) | 8.96 | 18.70 |
| GAD | 2.64x10^-3^ (2.51x10^-4^) | 1.57x10^-5^ (1.32x10^-6^) | 8418 (801) | 7.94 | 18.63 |
| AUDIT-C | 2.46x10^-3^ (2.13x10^-4^) | 1.48x10^-5^ (1.30x10^-6^) | 7856 (680) | 7.79 | 18.77 |
| CANN | 3.10x10^-3^ (4.82x10^-4^) | 1.03x10^-5^ (1.50x10^-6^) | 9889 (1536) | 7.66 | 17.38 |
| CUD | 1.93x10^-3^ (4.36x10^-4^) | 2.60x10^-5^ (5.20x10^-6^) | 6156 (1389) | 3.49 | 13.01 |
| MDD | 7.17x10^-3^ (9.08x10^-4^) | 5.26x10^-6^ (6.98x10^-7^) | 22862 (2896) | 2.14 | 12.53 |
| SWB | 7.75x10^-3^ (4.41x10^-3^) | 2.73x10^-6^ (1.10x10^-6^) | 24716 (14075) | 2.09 | 12.69 |
| ANX | 3.23x10^-2^ (2.85x10^-2^) | 2.60x10^-6^ (2.30x10^-6^) | 102890 (90951) | 1.92 | 11.79 |
| AGG | 4.43x10^-3^ (1.38x10^-3^) | 4.31x10^-6^ (1.01x10^-6^) | 14134 (4413) | 1.87 | 11.77 |
| AUDIT-P | 2.17x10^-3^ (3.21x10^-4^) | 1.17x10^-5^ (1.62x10^-6^) | 6931 (1025) | -0.02 | 10.96 |

^1^: number of causal variants explaining 90% of the h^2^_SNP_.

^2^: difference between the Akaike information criterion (AIC) or Bayesian information criterion (BIC) for the infinitesimal model assuming all genetic variants as causal and the best-fitting MiXeR model.

Supplementary Table 3. Bivariate MiXeR results for each of the 4 selected SUBs and SUDs and the 10 selected externalizing and internalizing traits.

| Trait 1 | Trait 2 | N unique causal variants for trait 1 (SD)^1^ | N unique causal variants for trait 2 (SD)^1^ | N shared causal variants (SD)^1^ | % of variants of trait 1 shared with trait 2 | % of variants of trait 2 shared with trait 1 | r_gs_ (SD)^2^ | r_g_ (SD)^3^ | Minimum AIC^4^ | Maximum AIC^5^ |
| --- | --- | --- | --- | --- | --- | --- | --- | --- | --- | --- |
| EverSmk | AFS | 689 (651) | 124 (179) | 10879 (245) | 94.0% | 98.9% | -0.6610 (0.0181) | -0.6376 (0.0072) | 133.04 | -0.05 |
|  | NSEX | 4329 (1218) | 3575 (1126) | 7239 (1046) | 62.6% | 66.9% | 0.8663 (0.1080) | 0.5516 (0.0114) | 1.83 | 3.61 |
|  | AFB | 1276 (769) | 109 (126) | 10292 (419) | 89.0% | 98.9% | -0.5367 (0.0151) | -0.5037 (0.0094) | 58.03 | -0.6 |
|  | RISK | 1274 (1553) | 1060 (1428) | 10294 (1746) | 89.0% | 90.7% | 0.3980 (0.1339) | 0.3384 (0.0136) | 7.61 | 0.17 |
|  | ADHD | 4171 (852) | 299 (295) | 7397 (455) | 63.9% | 96.1% | 0.7751 (0.0539) | 0.6061 (0.0151) | 3.18 | 0.01 |
|  | NEURO | 796 (664) | 1087 (593) | 10772 (566) | 93.1% | 90.8% | 0.1597 (0.0111) | 0.1468 (0.0105) | 42.54 | 0.09 |
|  | DEP-NEURO | 2075 (979) | 677 (823) | 9493 (758) | 82.1% | 93.3% | 0.3159 (0.0296) | 0.2751 (0.0148) | 13.85 | 0.14 |
|  | WORRY-NEURO | 259 (266) | 814 (781) | 11309 (533) | 97.8% | 93.3% | -0.0646 (0.0133) | -0.0617 (0.0127) | 91.98 | -0.3 |
|  | DEP | 315 (320) | 829 (438) | 11254 (557) | 97.3% | 93.1% | 0.3481 (0.0139) | 0.3315 (0.0136) | 20.92 | -0.76 |
|  | MOOD | 2077 (864) | 285 (398) | 9491 (474) | 82.0% | 97.1% | 0.2918 (0.0170) | 0.2603 (0.0120) | 15.87 | -0.3 |
| DrnkWk | AFS | 365 (314) | 3661 (632) | 7342 (635) | 95.3% | 66.7% | -0.2954 (0.0240) | -0.2346 (0.0137) | 47.23 | 0.58 |
|  | NSEX | 1056 (1472) | 4162 (1530) | 6651 (1492) | 86.3% | 61.5% | 0.5589 (0.1745) | 0.3800 (0.0156) | 4.18 | 0.99 |
|  | AFB | 318 (358) | 3012 (700) | 7390 (607) | 95.9% | 71.0% | -0.0490 (0.0219) | -0.0402 (0.0184) | 51.69 | -0.07 |
|  | RISK | 3701 (1746) | 7347 (1422) | 4007 (1618) | 52.0% | 35.3% | 0.8050 (0.2349) | 0.3054 (0.0184) | 0.45 | 2.47 |
|  | ADHD | 3296 (1031) | 3284 (959) | 4412 (1014) | 57.2% | 57.3% | 0.2789 (0.0974) | 0.1492 (0.0203) | 5.12 | 7.71 |
|  | NEURO | 252 (306) | 4403 (713) | 7456 (598) | 96.7% | 62.9% | -0.0234 (0.0225) | -0.0180 (0.0173) | 61.92 | 0.29 |
|  | DEP-NEURO | 509 (607) | 2971 (782) | 7199 (728) | 93.4% | 70.8% | -0.0394 (0.0216) | -0.0314 (0.0157) | 54.42 | 0.19 |
|  | WORRY-NEURO | 327 (441) | 4742 (810) | 7381 (590) | 95.8% | 60.9% | -0.0863 (0.0207) | -0.0656 (0.0147) | 36.31 | 0.2 |
|  | DEP | 410 (434) | 4785 (810) | 7298 (695) | 94.7% | 60.4% | 0.1078 (0.0211) | 0.0810 (0.0139) | 23.03 | -0.01 |
|  | MOOD | 489 (619) | 2557 (833) | 7219 (725) | 93.7% | 73.8% | -0.0345 (0.0259) | -0.0283 (0.0209) | 48.58 | -0.21 |
| Cigsperday | AFS | 55 (51) | 6764 (660) | 4226 (582) | 98.7% | 38.5% | -0.5691 (0.0579) | -0.3474 (0.0127) | 28.84 | 1.61 |
|  | NSEX | 50 (37) | 6585 (708) | 4231 (597) | 98.8% | 39.1% | 0.2518 (0.0292) | 0.1553 (0.0134) | 20.24 | 1.48 |
|  | AFB | 28 (28) | 6085 (777) | 4253 (576) | 99.4% | 41.1% | -0.6880 (0.0653) | -0.4365 (0.0197) | 13.01 | 0.46 |
|  | RISK | 384 (636) | 7462 (1142) | 3897 (975) | 91.0% | 34.3% | 0.4081 (0.1606) | 0.2091 (0.0194) | 4.55 | 1.16 |
|  | ADHD | 1068 (613) | 4509 (848) | 3213 (800) | 75.1% | 41.6% | 0.8308 (0.1544) | 0.4457 (0.0221) | 3.13 | 6.38 |
|  | NEURO | 128 (216) | 7745 (803) | 4153 (627) | 97.0% | 34.9% | 0.2320 (0.0400) | 0.1336 (0.0200) | 16.35 | 1.07 |
|  | DEP-NEURO | 1332 (1187) | 7186 (1300) | 2948 (1280) | 68.9% | 29.1% | 0.6686 (0.2798) | 0.2474 (0.0178) | 2.94 | 4.44 |
|  | WORRY-NEURO | 110 (84) | 8014 (793) | 4171 (584) | 97.4% | 34.2% | -0.0590 (0.0358) | -0.0338 (0.0203) | 46.21 | 1.57 |
|  | DEP | 473 (870) | 8228 (1024) | 3808 (943) | 88.9% | 31.6% | 0.4917 (0.1887) | 0.2398 (0.0211) | 4.23 | 0.99 |
|  | MOOD | 1146 (973) | 6656 (1364) | 3135 (1353) | 73.2% | 32.0% | 0.6132 (0.2695) | 0.2443 (0.0250) | 2.16 | 3.23 |
| AUD | AFS | 44 (44) | 3321 (407) | 7683 (340) | 99.4% | 69.8% | -0.6204 (0.0192) | -0.5166 (0.0075) | 26.85 | -0.92 |
|  | NSEX | 3364 (390) | 6451 (365) | 4363 (213) | 56.5% | 40.3% | 0.9534 (0.0415) | 0.4545 (0.0079) | -0.15 | 2.37 |
|  | AFB | 53 (38) | 2728 (485) | 7674 (343) | 99.3% | 73.8% | -0.4965 (0.0194) | -0.4248 (0.0097) | 24.82 | -0.85 |
|  | RISK | 3351 (860) | 6978 (898) | 4375 (729) | 56.6% | 38.5% | 0.7288 (0.1180) | 0.3320 (0.0123) | 0.59 | 1.62 |
|  | ADHD | 3093 (561) | 3062 (702) | 4633 (553) | 60.0% | 60.2% | 0.7923 (0.0895) | 0.4704 (0.0119) | 1.00 | 2.64 |
|  | NEURO | 424 (1026) | 4556 (1134) | 7302 (979) | 94.5% | 61.6% | 0.4242 (0.1297) | 0.3111 (0.0111) | 2.06 | -0.88 |
|  | DEP-NEURO | 1142 (1809) | 3585 (1725) | 6585 (1759) | 85.2% | 64.7% | 0.5176 (0.2272) | 0.3402 (0.0089) | 0.91 | -0.33 |
|  | WORRY-NEURO | 210 (153) | 4607 (688) | 7516 (341) | 97.3% | 62.0% | 0.3083 (0.0200) | 0.2393 (0.0149) | 4.29 | -0.90 |
|  | DEP | 3113 (292) | 7470 (468) | 4613 (149) | 59.7% | 38.2% | 0.9814 (0.0174) | 0.4688 (0.0088) | -0.58 | 3.59 |
|  | MOOD | 1095 (1822) | 3144 (1650) | 6631 (1766) | 85.8% | 67.8% | 0.5261 (0.2209) | 0.3575 (0.0126) | 0.65 | -0.76 |

^1^: number of causal variants explaining 90% of the h^2^_SNP_.

^2^: genetic correlation of shared variants.

^3^: genome-wide genetic correlation.

^4^: difference between the Akaike information criterion for the best-fitting MiXeR model and the minimum possible overlap restricted by the genetic correlation.

^5^: difference between the Akaike information criterion for the best-fitting MiXeR model and the maximum possible overlap restricted by the polygenicity of the least polygenic trait.

Supplementary Table 4. Univariate MiXeR results for Cigsperday excluding the cluster of nAchR genes at chromosome 15 and without excluding it.

| Trait | Discoverability (SD) | N of causal variants (SD)^1^ | AIC^2^ |
| --- | --- | --- | --- |
| Cigsperday excluding the cluster of nAchR genes at chromosome 15 | 2.44x10^-5^ (2.93x10^-6^) | 4281 (587) | 269.15 |
| Cigsperday with the cluster of nAchR genes at chromosome 15 | 5.96x10^-5^ (5.34x10^-5^) | 2285 (910) | 419.23 |

^1^: number of causal variants explaining 90% of the h^2^_SNP_.

^2^: difference between the Akaike information criterion (AIC) for the best-fitting MiXeR model and an infinitesimal model assuming all genetic variants as causal.
